# A rare case report of patent vitellointestinal duct with prolapsed orthograde ileal intussusception seen as bident horn

**DOI:** 10.1097/MS9.0000000000004997

**Published:** 2026-04-17

**Authors:** Jaya Ram Pandey, Prakash Kunwar, Suman Bikram Adhikari, Bal Mukund Basnet, Asmit Kumar Singh

**Affiliations:** a Department of Surgery, Nepalese Army Institute of Health Sciences, Kathmandu, Nepal; b Department of Pediatric Surgery, Kanti Children’s Hospital, Kathmandu, Nepal; c Janaki Medical College, Janakpur, Nepal

**Keywords:** bowel prolapse, case report, ileal intussusception, neonate, patent vitellointestinal duct, umbilical mass

## Abstract

**Introduction and importance:**

Patent vitellointestinal duct (PVID) with prolapsed bowel is an extremely rare congenital anomaly. Even rarer is the presentation with orthograde ileal intussusception.

**Case presentation:**

We report a 16-day-old neonate with a “Y”-shaped, red, mucosal mass protruding from the umbilicus, resembling a bident horn. Clinical examination revealed a prolapsed loop of ileum through a PVID without signs of obstruction or systemic sepsis. After stabilization with intravenous fluids, antibiotics, and thermal support, the patient underwent emergency laparotomy. Operative findings revealed prolapsed orthograde ileal intussusception through a PVID with unhealthy bowel. Resection of the duct and unhealthy segment was performed, followed by primary ileoileal anastomosis. The postoperative course was uneventful, and the infant remained asymptomatic at 3-month follow-up.

**Clinical discussion:**

Complete patency of VID with prolapsed orthograde intussusception is exceptionally rare, especially in neonates. The absence of systemic sepsis despite delayed presentation may be due to partial or intermittent intussusception with preserved bowel perfusion. The bident horn-like umbilical appearance represents a distinct clinical presentation. Surgical management requires prompt resection and anastomosis to prevent complications.

**Conclusion:**

This case highlights a unique presentation of PVID with orthograde ileal intussusception. Early recognition and timely surgical management are essential to achieve excellent outcomes, even in resource-limited settings.

## Introduction and importance

Remnants of vitellointestinal duct (VID) account for a wide variety of umbilical abnormalities. These include fistula, sinus tract, umbilical adenoma, enterocystoma and congenital bands^[^1^]^. Patent vitellointestinal duct (PVID) is reported to have incidence of 0.0053% with complete patency of VID being a rare incidence, and further rare is the condition where bowel is prolapsed through the patent duct^[^1–3^]^. Most cases of prolapsed ileum through a PVID occur in first 4 weeks of life but can occur from birth to 16 years of age^[^2^]^. Such condition has a male-to-female ratio of 4:1^[^2^]^.

Although remnants of the VID are not uncommon, complete patency with prolapsed orthograde intussusception is exceptionally rare. Previously reported cases of PVID with prolapsed bowel typically describe a singular tubular mucosal protrusion or inverted ileal loop emerging through the umbilicus. In contrast, our case demonstrated a distinctive bident horn-like morphology, where two limbs of the intussuscepted ileum appeared as Y-shaped mucosal projection at the limbs. This unusual appearance is a rare clinical manifestation of orthograde intussusception through a PVID and has not been clearly described in previous reports. This report also highlights the diagnostic and management challenges encountered in such a rare presentation in a resource-limited setting. This case report has been reported in line with the SCARE checklist^[^4^]^.

HIGHLIGHTS

- Patent vitellointestinal duct is a rare congenital anomaly.
- This case describes a bident horn-like structure at the umbilicus.
- The presentation was unusual in that the neonate had no systemic manifestations despite an unhealthy bowel.
- The condition was diagnosed clinically and managed surgically with an excellent outcome.
- Early recognition and timely surgical intervention are crucial to prevent complications.

## Case presentation

Sixteen-day-old male neonate presented with a progressively enlarging Y-shaped red mass emerging from the umbilicus. Initially, it started as a small swelling (size of pea) few days after separation of umbilical cord and gradually enlarged, especially during crying. There was no history of discharge of pus, bile, or fecal matter through the umbilicus. He was passing normal stool per rectally, and there were no signs of obstruction or systemic infection. The patient’s family history and allergic history were unremarkable.

**Figure 1.**
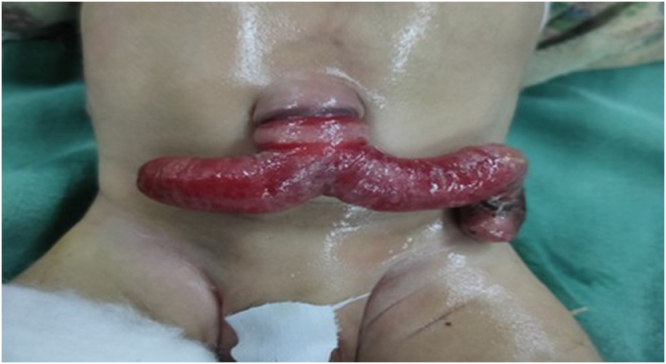
Bident horn in anterior abdomen of neonate.

On examination, a red colored, Y-shaped mucosal loop was noticed emerging from anterior abdominal wall through umbilicus with dusky color at one end and was irreducible and bled on touch, which suggested intestinal mucosal surface (Fig. 1). There were no features of dehydration or sepsis/septic shock (hypotension, tachycardia, decreased urine output). Rest of the systemic examinations were unremarkable. No other congenital abnormalities were detected.

The neonate was resuscitated with intravenous fluids, administered broad-spectrum antibiotics, and kept warm. Nasogastric decompression was performed although there was no obstruction, and prolapsed bowel was covered with warm saline-soaked gauze. Routine hematological and biochemical parameters were within normal range. Ultrasound examination of the abdomen was absolutely normal.

**Figure 2.**
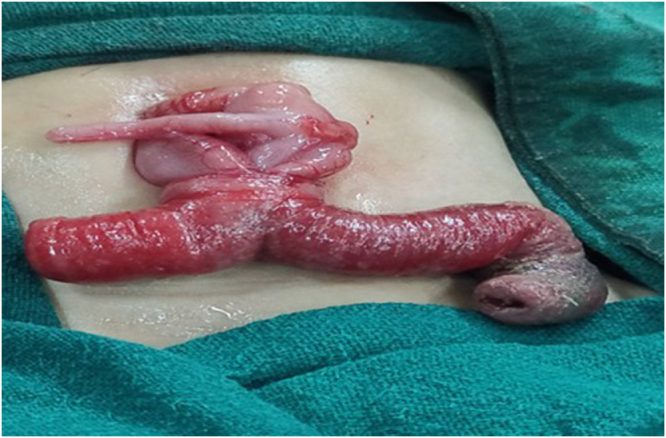
Bident horn-like mucosal mass protruding from the umbilicus, representing prolapsed ileal loops through a PIVD. The two protruding limbs give a characteristic Y-shaped appearance, caused by orthograde ileal intussusception through the patent duct. The prolapsed bowel appears edematous with areas of discoloration, suggesting compromised bowel viability.

**Figure 3.**
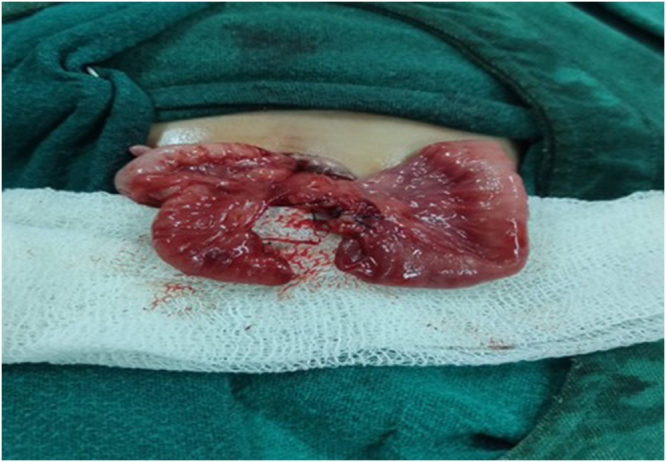
Intraoperative findings showing small bowel after resection and ready for anastomosis.

Emergency laparotomy via a periumbilical incision revealed a prolapsed orthograde ileal intussusception through a PVID (Fig. 2). Non-operative management or delayed surgical intervention was considered inappropriate due to the irreducible nature of the prolapsed bowel through a PVID, evidence of mucosal compromise, and the potential for rapid deterioration. Approximately 8 cm of unhealthy ileum, including the duct, was resected (Fig. 3), and primary ileo-ileal anastomosis was performed. Primary anastomosis was chosen because the remaining bowel margins were healthy and well vascularized after resection of the affected segment. In neonates with stable hemodynamic status and viable bowel edges, primary anastomosis is preferred as it restores bowel continuity and avoids the morbidity associated with stoma formation and subsequent reversal procedures. The postoperative course was uneventful. The neonate received parenteral nutrition for 48 hours and was discharged on postoperative day 7 in good condition. At 3-month follow-up, the infant remained asymptomatic with appropriate weight gain (Table 1).

**Table 1.** Timeline of events.

Day	Clinical event
Day 5 after cord separation	Small umbilical swelling noticed by parents
Day 10	Progressive enlargement of umbilical mass and seek medical attention, referred to higher Centre
Day 16	Presentation to hospital with Y-shaped mucosal mass; clinical diagnosis and stabilization; emergency laparotomy performed
Postoperative Day 7	Discharged in good condition
3-month follow-up	Asymptomatic with appropriate growth

## Clinical discussion

Around 5 weeks of gestation, the midgut is herniated into umbilical cord and returns to abdominal cavity at the 10th week of gestation^[^5^]^. VID, in a developing embryo, is a bridge between yolk sac and the gut which tends to obliterate in the 5th–7th weeks of gestation^[^1,6^]^. Remnants of VID due to failure of involution include abnormalities like Meckel’s diverticulum (2–3%), patent duct, solid cord, central cyst, diverticulum, or a rare condition of bowel prolapsed^[^1,7^]^. Meckel’s diverticulum inversion is common cause of intussusceptions in VID cases^[^3,8^]^

These generally present as umbilical discharge of mucus, flatus, feces, or mucosal prolapse through the umbilicus^[^9^]^. Ileum prolapses through the patent duct after an episode of crying or coughing^[^2,10^]^. Intussusception of small bowel in VID is a rare context and can be explained since the VID and ileocecal valve are nearer in infants causing higher intraluminal pressure in the presence of wide mouth and short duct^[^6^]^. Intussusception can be prograde (proximal limb), retrograde (distal limb), or orthograde (both limbs)^[^9^]^. Double intussusceptions of small bowel through PVID are even a rarer condition^[^10,11^]^.

VID is also generally associated with umbilical hernia, volvulus neonatorum, congenital heart malformation, cleft lip, cleft palate, and spina bifida^[^2^]^.

Prolapse of bowel through the PVID is a serious complication. Such conditions require prompt surgical intervention with primary closure and reduction of prolapsed intestines if presented without gross edema but can be approached with exteriorization of suspected loop or loop ileostomy when there is gross edema and uncertain viability of intestinal loops^[^1,3,5^]^. In 2021, Rajendra K. Ghritlaharey^[^12^]^ suggested that the surgical resection of PVID can be carried out either through resection of PVID along with wedge resection of ileum and ileal repair or by resection of PVID with adjacent small segment of ileum and ileo-ileal anastomosis, the later to be the preferred route^[^12^]^.

Also, there have been 8.3% cases of mortality seen in infants with postoperative complications like anastomotic leak, septicemia, peritonitis, and postoperative intestinal obstruction or adhesion^[^12^]^. The prognosis is usually worse if it presents with prolapsed ileum through the duct^[^2^]^.

Interestingly, despite the presence of an unhealthy bowel segment, the neonate did not develop systemic sepsis. This is explained by intermittent or partial intussusception, where vascular compromise occurs gradually without complete obstruction of blood flow. Such partial perfusion may delay progression to bowel necrosis and systemic inflammatory response, explaining the absence of septic manifestations at presentation. Similar cases of bowel prolapse through a PVID have been reported, although the clinical presentation and management vary^[^1,5,9^]^. Mohite *et al* described a 5-month-old infant with an inverted ileal loop prolapsing through a patent duct. Due to significant bowel edema, the patient was treated with resection of the duct and creation of a loop ileostomy, which was later reversed. Lone *et al* reported a case of double prolapse of the ileum through a PVID associated with omphalocele, managed by reduction of the prolapsed bowel loops, resection of the duct, and repair of the abdominal wall defect. However, early diagnosis and prompt surgical intervention are critical to prevent necrosis and sepsis.

Management of rare neonatal surgical emergencies in resource-limited settings presents additional challenges. Limited access to advanced diagnostic imaging and delays in referral or transportation often complicate timely diagnosis and treatment. In our case, the patient had been referred from a primary health care center 5 days prior but reached our tertiary center only after this delay. Although computed tomography imaging was initially planned, it could not be performed due to resource limitations and the urgency of the condition. Therefore, the diagnosis relied primarily on careful clinical examination. This case highlights that even in resource-constrained environments, timely recognition and appropriate surgical management can lead to favorable outcomes.

Differential diagnosis: Several conditions may present as an umbilical mass in neonates and were considered in this case. Umbilical granuloma typically presents as a small, moist, pink lesion with serous discharge after cord separation and lacks mucosal characteristics. Umbilical polyps are firm, reddish nodules composed of intestinal mucosa but usually do not demonstrate a prolapsed intestinal loop or increase in size during crying. Omphalocele minor is characterized by herniation of abdominal contents through the umbilical ring covered by a membranous sac, which was absent in this case. The presence of a mucosal Y-shaped prolapsing structure that bled on touch strongly suggested intestinal mucosa, favoring a diagnosis.

Limitations: Short follow-up duration and lack of proper preoperative imaging and evaluation

## Conclusion

PVID may rarely present with prolapsed orthograde ileal intussusception. Even in the absence of sepsis or obstruction, early recognition and surgical management are essential to prevent serious complications. This case emphasizes the importance of clinical vigilance and prompt surgical intervention in neonates presenting with unusual umbilical masses, particularly those without systemic manifestations.

## Data Availability

The data supporting the findings of this study are available from the corresponding author upon reasonable request.

## Research registration unique identifying number (UIN)

Not applicable. This is a case report.

## Peer and provenance statement

Not commissioned, externally peer reviewed.
